# Laser Scribed Graphene Cathode for Next Generation of High Performance Hybrid Supercapacitors

**DOI:** 10.1038/s41598-018-26503-4

**Published:** 2018-05-25

**Authors:** Seung-Hwan Lee, Jin Hyeon Kim, Jung-Rag Yoon

**Affiliations:** 1 Institute for Research in Electronics and Applied Physics, University of Maryland, College Park, MD 20742 USA; 2 Department of Electronics Materials Engineering, Kwangwoon University, Seoul, Korea; 3 R&D Center, Samwha Capacitor, Yongin-si, Gyeonggi-do Korea

## Abstract

Hybrid supercapacitors have been regarded as next-generation energy storage devices due to their outstanding performances. However, hybrid supercapacitors remain a great challenge to enhance the energy density of hybrid supercapacitors. Herein, a novel approach for high-energy density hybrid supercapacitors based on a laser scribed graphene cathode and AlPO_4_-carbon hybrid coated H_2_Ti_12_O_25_ (LSG/H-HTO) was designed. Benefiting from high-energy laser scribed graphene and high-power H-HTO, it was demonstrated that LSG/H-HTO delivers superior energy and power densities with excellent cyclability. Compared to previous reports on other hybrid supercapacitors, LSG/H-HTO electrode composition shows extraordinary energy densities of ~70.8 Wh/kg and power densities of ~5191.9 W/kg. Therefore, LSG/H-HTO can be regarded as a promising milestone in hybrid supercapacitors.

## Introduction

**Figure 1:**
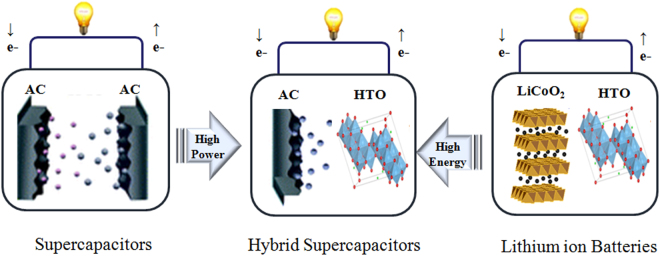
Concept of hybrid supercapacitors. Hybrid supercapacitors are comprised of supercapacitor-type cathode (AC) and battery-type anode (HTO). This is well-balanced system with a highly accelerated Li-ion intercalating HTO anode and a non-faradaic AC cathode using an anion adsorption-desorption process.

Hybrid supercapacitors were proposed by Naoi in 2009 in an attempt to maximize the benefits of existing supercapacitors (high power density and stable cycle performance) and lithium-ion batteries (high energy density) by using an asymmetric electrode^1^. Conventional supercapacitors consist of symmetrical electrodes made of activated carbon, having capacity of ~30 mAh g^−1^ ^2^. Alternatively, hybrid supercapacitors are composed of metal oxide anodes derived from lithium-ion batteries and carbon-based cathodes derived from supercapacitors, as shown in Fig. 1 ^1^. The capacity of a metal oxide anode is several times higher than that of an activated carbon anode. Therefore, hybrid supercapacitors can deliver higher energy densities than supercapacitors^3^. Many researchers have studied hybrid supercapacitors as next-generation energy storage devices, but there are still some problems that need to be addressed; these include increasing the energy and power densities, cycle life, and reliability^4^. The most important factor determining the performance of hybrid supercapacitors is their electrodes^4,5^. There are currently three main research directions to improve electrodes for high-performance hybrid supercapacitors: (i) improving the power density and cycle life of the anode, which are drawbacks of lithium-ion battery-type electrodes; (ii) suppressing the swelling for higher reliability and safety; and (iii) improving the energy density of the cathode^6–9^. One of the most important goals of hybrid supercapacitor research is to increase the energy density. In order to improve the energy density of hybrid supercapacitors, an effective method is to increase the capacitance of the carbon-based cathode, which is much lower than that of the metal oxide anode. To do this, it is necessary to replace activated carbon with graphene. Graphene supercapacitors have been reported to deliver capacitances of 200–300 F g^−1^, which are higher than those of AC supercapacitors (∼100 F g^−1^)^10^.

Graphene, which is a monolayer of carbon atoms tightly packed into a two-dimensional (2D) honeycomb lattice, can be regarded as the basic building block of all graphitic materials. For example, zero-dimensional fullerenes, one-dimensional carbon nanotubes (CNTs), and three-dimensional graphite can be prepared by wrapping, rolling, and stacking graphene, respectively^11^. Since the first report of graphene by Geim and Novoselov in 2004, many researchers have attempted to apply graphene to high-performance energy storage devices^12,13^. In particular, many researchers have attempted to use graphene in energy storage devices (supercapacitors, batteries, fuel cells, and solar cells), electronics, and biomedical devices, largely due to its extraordinary electronic, mechanical, and physicochemical properties^14,15^. These advantageous properties are due to the fact that graphene has a large specific surface area (2630 m^2^/g), high capacitance (~550 F/g), good electrical conductivity (~1 × 10^3^ S/cm), and good mechanical strength (42 N/m)^15–17^.

There are three main reduction methods that can be used to prepare graphene from graphite oxide (GO). The first is the micromechanical cleavage (MMC) method, which is a simple procedure that isolates graphitic layers from highly oriented pyrolytic graphitic (HOPG) by using adhesive tape^18^. However, this approach is time consuming and difficult to reproduce on a massive scale^19^. Moreover, it yields randomly distributed graphene sheets with uncontrollable morphologies^20^. The second method is epitaxial growth on single-crystalline silicon carbide (SiC). Although this method does produce high-quality, large-area graphene, it is limited by strict processing conditions, such as its high temperature, ultrahigh vacuum, and the use of SiC; this results in uncompetitive prices and complex fabrication procedures^21,22^. Finally, liquid-phase exfoliation is a cost-effective method that uses organic solutions. However, despite its advantages, this approach has the following drawbacks: (1) it is difficult to remove the surfactants used during fabrication, (2) there is a reduction in the graphene sheet size due to the excessive sonication process, (3) graphene aggregation occurs via the strong tendency of π-π stacking between chemically reduced graphene sheets, and (4) it requires a high boiling point and an expensive exfoliation medium in order to produce high-quality graphene^23–26^. As a result, all three of these methods remain far from commercialization.

Recently, graphene synthesis via laser scribing (LSG) has been spotlighted as a one-step fabrication approach. LSG can be obtained by the simultaneous reduction and exfoliation of graphite oxide, without a reducing agent^27–32^. Most importantly, the as-prepared graphene can be used directly as an electrode, which means that the aggregation of LSG can be effectively prevented because there is no additional processing. Additionally, LSG can be used without a binder or a conductive additive, both of which have negative effects on the capacity per unit weight.

In this paper, we demonstrate how the electrochemical performance of LSG is superior to conventional activated carbon. Moreover, to evaluate the practical application of LSG, LSG cathode-based hybrid supercapacitors with an AlPO_4_-carbon hybrid-coated HTO anode (H-HTO) were made; this anode was previously shown to demonstrate excellent electrochemical performance^33^. This novel system outperforms previously reported hybrid supercapacitors in terms of the energy and power densities, benefiting from the remarkable advantages of high-energy LSG and high-power, long-life H-HTO. These findings suggest a new way to fabricate advanced hybrid supercapacitors.

### Experiments

**Figure 2:**
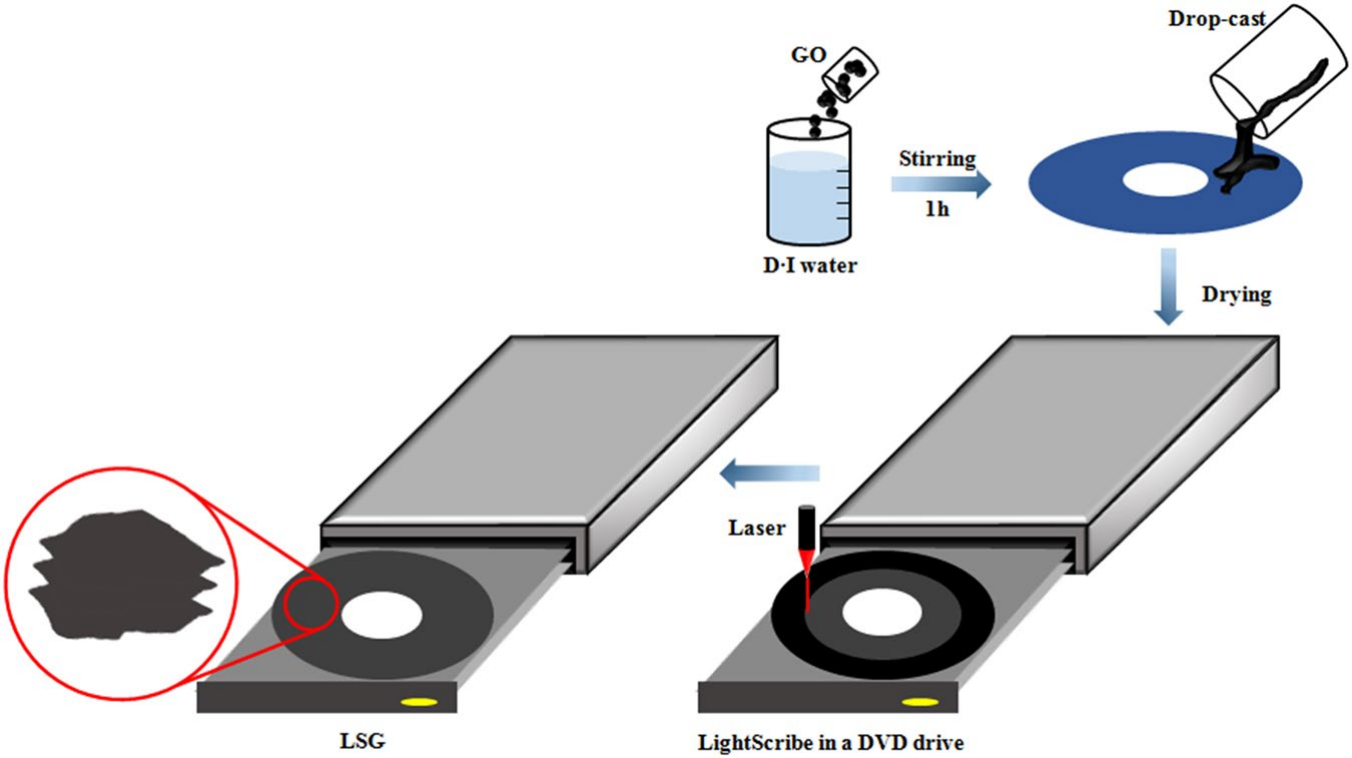
Schematic illustration for the preparation process for LSG. The GO was first suspended in DI water. Then, solution was dropped onto aluminum foil that was glued to the surface of a DVD disc. The GO-coated DVD disc was then inserted into the DVD optical drive for laser treatment.

LSG was prepared using a DVD burner (Hitachi/LG GH40L; 788-nm laser with a maximum power of 5 mW). GO was prepared via a modified Hummers’ method. The mixture of GO in an aqueous solution was dispersed by ultrasonication for 180 min, and then the homogeneous solution was dropped directly onto aluminum foil that was glued to a DVD disc for laser treatment. After drying under ambient conditions for 24 h, the dried film was inserted into a DVD burner to prepare LSG for 5 h. LSG can be used directly as a cathode for hybrid supercapacitors without the addition of a binder or conductive additives, as shown in Fig. 2. An AC electrode was fabricated by mixing AC (MSP-20, 90 wt%) with conductive carbon (5 wt%) and polytetrafluoroethylene (PTFE, 5 wt%). The thicknesses of LSG and AC were 150 μm and 100 μm, respectively. The LSG can have a larger thickness compared to AC. This is because LSG exhibits high capacitance as well as good performance especially under high current density due to higher effective surface area. On the other hand, AC has a high electrical resistance, resulting from limited diffusion of ions in the inner porous network of the activated carbon. It limits the thickness and generally uses conductive materials (such as carbon black) with high conductivity and low surface area for fast electrical charge transfer. The H-HTO anode was also prepared as previously reported^33^. The structural properties were analyzed using X-ray diffraction (XRD), transmission electron microscopy (TEM), selected area electron diffraction patterns (SAED), energy-dispersive X-ray spectroscopy (EDS) mapping, and Raman spectroscopy. The surface area of LSG was measured with a Quantachrome Autosorb-3b Brunauer–Emmett–Teller (BET) surface analyzer, and the electrical conductivity of LSG was measured by the four-point probe method at room temperature.

To carry out the half-cell test, electrodes were prepared with a Li-metal counter electrode and separators. Coin-type hybrid supercapacitors were fabricated in an Ar-filled glovebox. Before being impregnated with a 1.5 M LiPF_6_ solution in 1:1 ethylene carbonate (EC):dimethyl carbonate (DMC), which was used as the electrolyte, the fabricated cell was dried in a vacuum oven for 48 h to remove moisture. Finally, the LSG cathode, H-HTO anode, and separator were assembled in an Ar-filled glovebox to carry out the full-cell test. The galvanostatic charge-discharge curves, rate capabilities, and cycle performances were measured using an Arbin BT 2042 battery test system. The cyclic voltammograms (CVs) were measured using a potentiostat (Iviumstat), and electrochemical impedance spectroscopy (EIS) was conducted using a CHI660D electrochemical workstation in the frequency range of 10^−1^ to 10^6^ Hz.

## Results and Discussion

**Figure 3:**
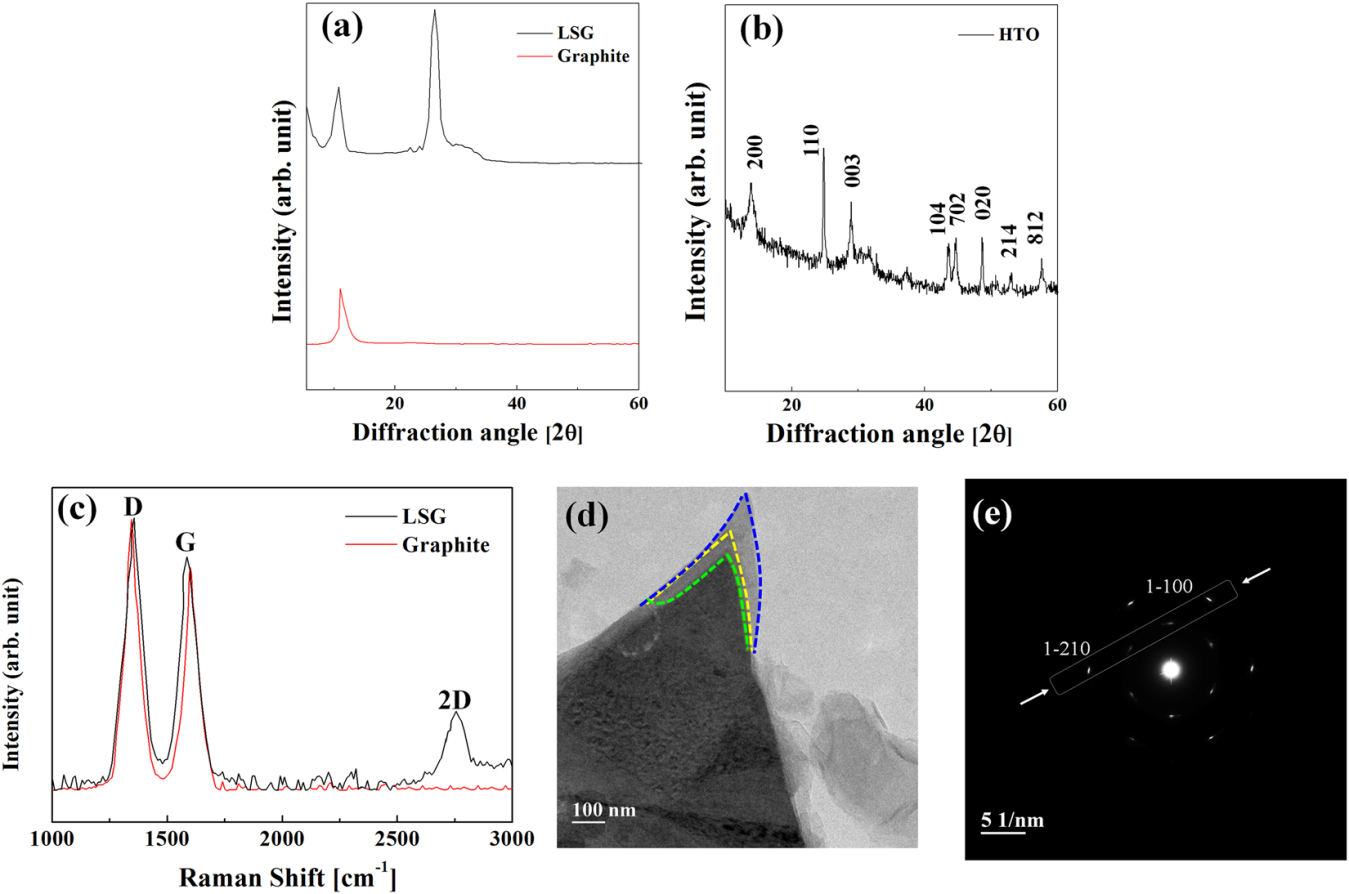
Structural and morphological characterization of as-synthesized LSG, GO and H-HTO. (**a**) XRD patterns of LSG and GO. The diffraction peak (2θ = 26.44°) indicates that GO is reduced to LSG (**b**) XRD patterns of HTO (**c**) Raman spectra of LSG and GO. There are D peak at 1355 cm^−1^, G peak at 1586 cm^−1^ and 2D peak at 2755 cm^−1^, which corresponds to graphene. (**d**) TEM image of LSG, showing its three layers (**e**) SAED patterns of the LSG where a few typical Bragg reflections are also indexed.

The chemical compositions of the as-prepared LSG and H-HTO were analyzed by looking at the XRD patterns, as shown in Fig. 3(a,b). The XRD peaks of LSG are different from GO, having only one diffraction peak (10.75° 2θ). A GO peak is still observed in the LSG, indicating that the upper part of GO is reduced to LSG while the lower part remains as GO^34,35^. The diffraction peaks of H-HTO can be indexed as (200), (110), (003), (104), (702), (020), (214), and (812) with the space group *P2/m*, which belong to HTO^36^. No characteristic peaks from any other impurities were observed in the XRD patterns, indicating the high purity of the materials^37^. This is related to the very low content of coating materials, which is below the instrument detection limit^38^. The TEM image and EDS mapping of H-HTO are shown in Figure S1, allowing us to confirm that H-HTO is well formed.

The formation of LSG was further confirmed by Raman spectroscopy, which allows us to investigate the structure of graphite-based materials. The Raman spectra were measured to further demonstrate the reduction of graphite oxide to graphene via laser scribing, as shown in Fig. 3(c). Both LSG and GO have high G and D peaks; the biggest difference between LSG and GO is the presence of a 2D peak. There is a 2D peak in LSG, indicating the formation of LSG generated by the laser scribing process, while no 2D peak is present in the GO spectrum^39^. The D and G peaks are mostly related to the first-order Raman scattering phonon vibrational mode and the sp2-hybridized bonded carbon atoms in the two-dimensional hexagonal lattice, respectively^40^. The 1345 cm^−1^ and 1605 cm^−1^ peaks can be assigned to the D peak and G peak for GO. Alternatively, LSG displays a disorder-induced D peak at 1355 cm^−1^, indicating a breathing mode of k-point photons for A_1g_. The G peak at 1586 cm^−1^ is also observed, indicating the first-order scattering of the E_2g_ mode^41^. Although the D peak of LSG is slightly narrowed, it is not significantly changed. GO is a carbon lattice containing oxygen functional group defects, which break carbon-carbon bonds to form a strong D peak. The carbon-oxygen functional group bonds break and oxygen is released during the laser scribing process. C-C-recombination is difficult because of the thickness expansion caused by loose LSG, which results from laser scribing^42^. The G peak of LSG is shifted to a lower wave number, compared to GO, due to the reduced oxygen functional groups^43^. Moreover, the strong 2D peak at 2755 cm^−1^ is a result of the reduction of GO by the laser scribing process. Additionally, the I_2D_/I_D_ ratio was 0.36 with a full width at half maximum (FWHM) of 66 cm;^−1^ this value corresponds to three graphene layers^44,45^.

In order to examine more structural information related to the as-prepared LSG, TEM and selected area electron diffraction pattern (SAED) analyses were conducted, as shown in Fig. 3(d,e) respectively. We can clearly see that the as-prepared LSG consists of three layers of graphene. The stacking of LSG was further investigated by analyzing the line profiles of the SAED patterns^46^. Although the SAED patterns for graphene provide the same hexagonal structure regardless of the number of layers, the number of layers can be determined by analyzing the relative intensity^47^. The (1–210) intensity was 48% higher than the (1–100) intensity, according to the Bravais-Miller (hkil) indices. As reported previously, the intensity ratio of the outer and inner peaks for tri-layer graphene is known to be about 1.48^47,48^, which is consistent with our value. The intensity ratio of the outer peak equivalent planes I(1–210) to the inner peak from I(0–110) for LSG was 1.55. The presence of three graphene layers in LSG can be explained by the stacking or aggregation of graphene layers during laser scribing^49^. TEM and SAED analyses are consistent with the Raman spectra, indicating the tri-layer nature of LSG.

Figure S3 shows the N_2_ adsorption–desorption isotherms of LSG. The BET measurements show that LSG has a specific surface area of 573.6 m^2^ g^−1^ with a conductivity of 1529 S m^−1^. A hysteresis loop in the N_2_ adsorption/desorption isotherms of LSG was also observed, indicating its porous structure^50^. It is well known that a large surface area is the most important factor for high capacitance in carbon-based electrodes; this property is related to a higher ion-accessible surface area between the electrode and electrolyte interface, resulting in outstanding electrochemical performances^51,52^.

**Figure 4:**
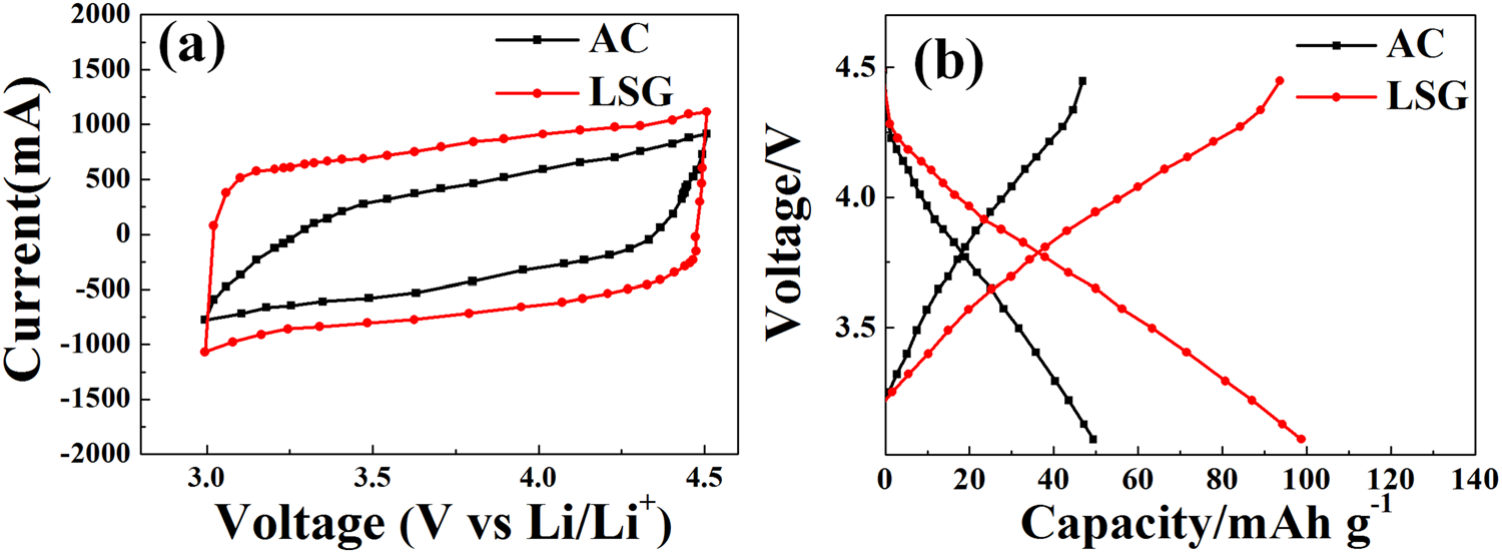
Electrochemical performance of the LSG and AC cathode in a half-cell system. (**a**) CV curves at a scan rate of 50 mV/s and (**b**) initial charge-discharge curves at a current density of 0.5 A/g. For half-cell tests, cells were assembled into two-electrode configuration with a Li metal counter electrode, a separator, and an electrolyte a 1.5 M lithium Hexafluorophosphate in a 1:1 mixture of ethylene carbonate and dimethyl carbonate in a glove box.

Figure 4(a) shows the cyclic voltammetry (CV) curves of LSG and activated carbon (AC). LSG shows a wider area, which means more electrochemical reactions in its charge-discharge process. More importantly, LSG can preserve a rectangular shape under a high scan rate of 50 mV/s, while a distortion in the shape was observed for AC, clearly indicating the superior rate capability of LSG^53^. We also demonstrated that LSG delivers a higher capacity than AC (Fig. 4(b)). This is because LSG has a high conductivity and high effective specific surface due to the prevention of graphene aggreg

w typical hybrid supercapacitor shapes with a couple of broad redox peaks (an oxidation peak of 2.8 V and a reduction peak of 2.61 V), demonstrating that hybrid supercapacitors possess redox reactions and capacitive characteristics. The polarization value can be obtained via the followination. As a result, LSG has superior capacitive behavior compared to AC. The capacity value mentioned in Fig. 4(b) is taken from the first cycle. Because LSG and AC have excellent electrode stability and cycle performance based on the same energy storage mechanism, there is not a significant difference in their capacity at an increased number of cycles. Therefore, it is important to measure the capacity in the first cycle.

**Figure 5:**
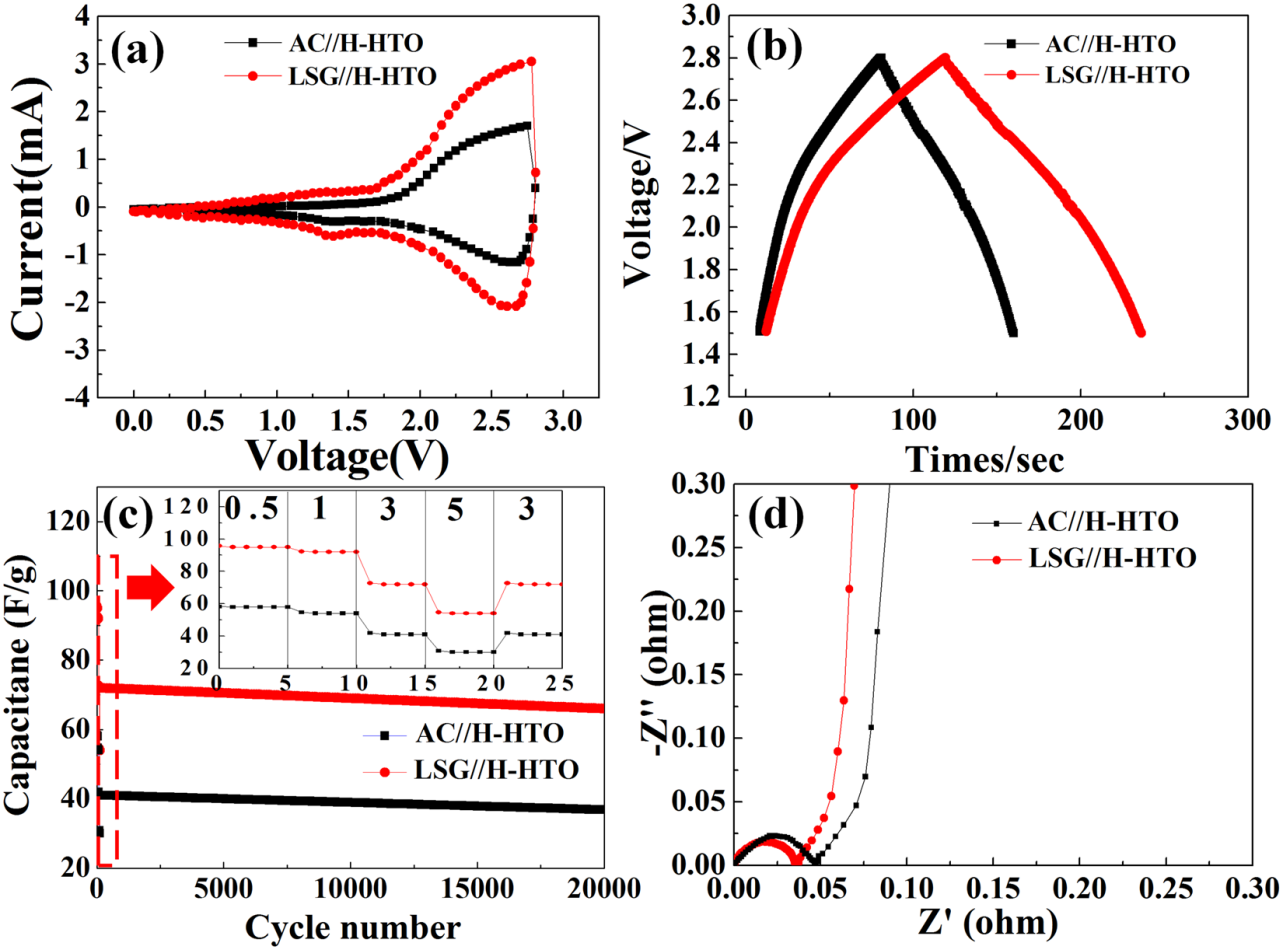
Electrochemical performance of hybrid supercapacitors based on LSG cathode and H-HTO anode in a full-cell system. (**a**) CV curves in the voltage of 0–2.8 V at a scan rate of 0.1 V/s and (**b**) initial charge-discharge curves in the voltage of 1.5–2.8 V at a current density of 0.5 A/g_cathode+anode_ (**c**) long-term cycling performance of hybrid supercapacitors at a current density of 3 A/g (the inset shows the rate capability at various current rates from 0.5 A/g to 5 A/g) (**d**) Nyquist curves for the frequency range from 10^−1^ to 10^5^ Hz.

Figure 5(a) shows the CV curves from 0 to 2.8 V, which can be used to better understand the electrochemical reactions of hybrid supercapacitors. Both CV curves show typical hybrid supercapacitor shapes with a couple of broad redox peaks (an oxidation peak of 2.8 V and a reduction peak of 2.61 V), demonstrating that hybrid supercapacitors possess redox reactions and capacitive characteristics. The polarization value can be obtained via the following equation^54^:

1 $${\rm{\Delta }}{\rm{E}}={{\rm{E}}}_{{\rm{a}}}-{{\rm{E}}}_{{\rm{c}}}$$

Here, *ΔE* is the polarization, *E*_*a*_ is the anodic peak, and *E*_*c*_ is the cathodic peak. It can be seen that the polarization value of hybrid supercapacitors is sufficiently small (0.12 V), demonstrating the high reversibility of LSG and H-HTO^55–57^. From the polarization value, the lithium ion insertion/extraction efficiency during the charge-discharge process can be deduced. The polarization can influence the electrochemical performances of hybrid supercapacitors, including the energy and power densities, rate capability, and cycle performance.

Figure 5(b) shows the initial charge-discharge curves of the hybrid supercapacitors using LSG and the AC cathode at a current density of 0.5 A/g. All hybrid supercapacitors exhibit asymmetric and non-linear charging profiles, which are typical of hybrid supercapacitors. The discharge time of LSG is longer than that of AC. The specific discharge capacitances of a hybrid supercapacitor can be estimated by referencing the following equation^4^:

2 $${\rm{C}}=\frac{q}{{\rm{\Delta }}V\times m}=\frac{\int i{\rm{\Delta }}t}{{\rm{\Delta }}V\times m}.$$

Here, *i* is an absolute value of the charge and discharge current, *t* is the time during the discharge process, *V* is the potential difference of discharging, and *m* is the mass of active materials. According to CV analysis, LSG exhibited an initial capacitance of 95.7 F/g, which was higher than that of AC (58.2 F/g). This is due to the high effective specific surface area of LSG. Also, it can be clearly seen that both hybrid supercapacitors have an IR drop (I = current, R = resistance) when charging is complete and discharging begins; this can be explained by the fact that the polarization is closely associated with slow lithium ion movement during lithium intercalation in the transition-metal oxide electrode. The IR drop can be calculated via the following equation^58^:

3 $$R=\frac{{V}_{charge}-{V}_{discharge}}{2l}.$$

Here, *V*_*charge*_ is the voltage of the cell at the end charge, *V*_*discharge*_ is the voltage of the cell at the starting discharge, and *I* is the absolute value of the charge and discharge current. The calculated IR drops of LSG and AC are 0.2 and 0.26 Ω, respectively, suggesting a very low value for the hybrid supercapacitors^59,60^.

The inset of Fig. 5(c) shows the retention of hybrid supercapacitors as a function of the current density. As expected, the hybrid supercapacitors displayed a higher retention when using LSG (91.92%) instead of AC (88.69%). Moreover, as the current density increased from 0.5 to 5.0 A/g, the difference between the LSG and AC supercapacitors increased. At a current density of 5.0 A/g, the retention of LSG remained at 63%, which was much higher than that of AC (31% retention). As a result, we conclude that the advantages of LSG are more noticeable at greater current densities. More significantly, after returning to 3.0 A/g, the capacitance recovers to its original state completely, demonstrating that LSG/H-HTO compositions are stable and reversible^61,62^. For LSG, more ions can contribute to the capacitive behavior to maintain the retention, as compared with AC. This is primarily due to the higher conductivity and shorter pathways for ion diffusion from the electrolyte. After returning to 3.0 A/g, the cycling performances of the hybrid supercapacitors were analyzed (Fig. 5(c)). Both hybrid supercapacitors showed outstanding long-term cyclic performances. The retention of LSG was about 90.7% after 20000 cycles, without notable capacity fading, indicating the remarkable stability that is caused by the synergistic effect of LSG/H-HTO. In other words, this excellent cyclability may be ascribed to the high capacitance and retention of LSG, which play an important role in providing stable, high capacitance to the hybrid supercapacitor. In addition, H-HTO contributes to the rate capability and cycle performance of the hybrid supercapacitors due to the formation of efficient and rapid pathways, which allow for smooth Li ion diffusion (AlPO_4_) and electron transport (carbon)^33^. The rate performances of five LSG/H-HTO hybrid supercapacitors were measured to verify their reproducibility, as shown in Figure S2. It can be confirmed that the improvement in performance is accompanied by excellent reproducibility in the LSG/H-HTO hybrid supercapacitors.

Nyquist plots obtained from the EIS of the hybrid material are shown in Fig. 5(d). The semicircle in the middle frequency is referred to as the charge transfer resistance (R_ct_) and is related to the interfacial resistance between the electrode and electrolyte. It is obvious that both R_ct_ values are small, with LSG (0.036 Ω) showing a slightly lower resistance than AC (0.047 Ω). This observation can be ascribed to the hybrid coating of HTO, which acts as a bridge for smooth Li ion and electron transfer.

**Figure 6:**
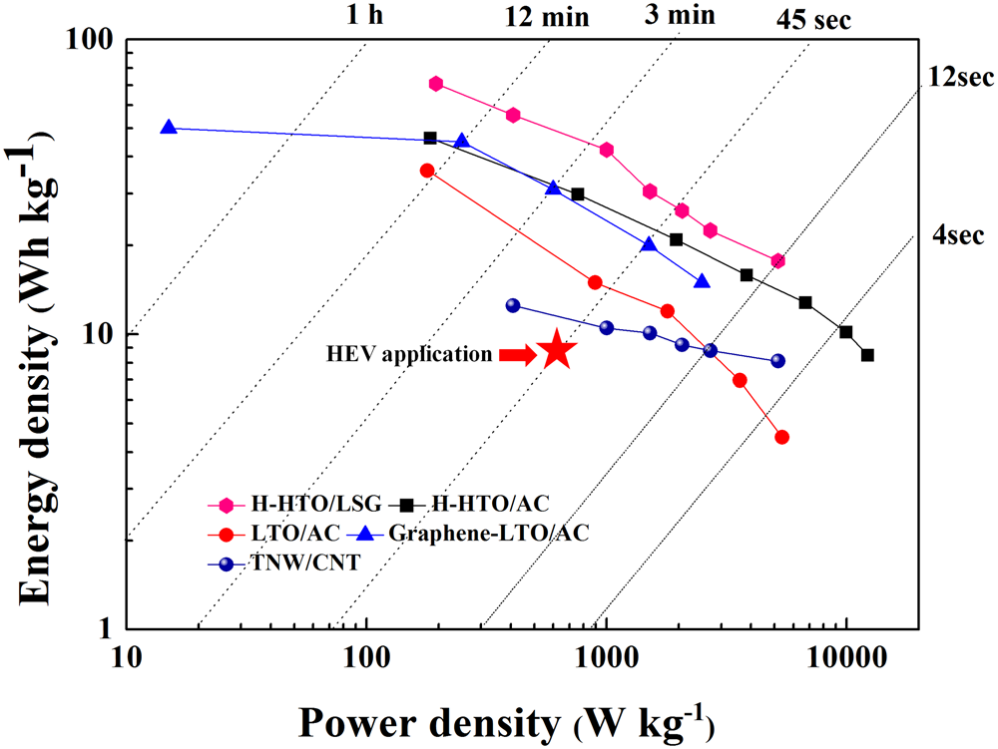
Energy and power densities of LSG/H-HTO hybrid supercapacitors compared with previously reported hybrid supercapacitors. LSG/H-HTO hybrid supercapacitors delivered dramatically improved energy and power densities. LSG/H-HTO hybrid supercapacitors are compared with other hybrid supercapacitors using H-HTO/AC^63^, LTO/AC^64^, TNW/CNT^65^ and Graphene-LTO/AC^66^.

The specific energy and power densities of hybrid supercapacitors are shown in Fig. 6. The power density (*P*) and energy density (*E*) were calculated as follows^4^:

4 $$E=1/2{{\rm{C}}}_{{\rm{cell}}}{{\rm{V}}}^{2}$$

5 $$P={\rm{E}}/{\rm{\Delta }}{\rm{t}}$$

Here, *E* is the energy density, *C*_*cell*_ is the specific capacitance of the hybrid supercapacitor, *V* is the voltage, *P* is the power density, and *Δt* is the discharge time. LSG provides an energy density between 17.7 and 70.8 Wh/kg and a power density between 195.1 and 5191.9 W/kg, both of which are greater than those of H-HTO/AC, Li_4_Ti_5_O_12_ (LTO)/AC, graphene-LTO/AC, or titanate nanowire (TNW)/CNT^63–65^. These energy and power densities also fully meet the requirements of hybrid electric vehicle (HEV)^66^. Furthermore, compared to other previously reported hybrid supercapacitors, LSG is shown to be superior. LSG can result in thicker electrodes than AC, ensuring higher capacitance per area. This is the case because AC exhibits a higher electrical resistance. Since the entire thickness of AC does not react under fast charging and discharging, and only the upper part of AC contributes to the capacity, the thickness of AC is limited. In addition, AC requires the use of carbon black (Super P), which has a small specific surface area and high conductivity; additionally, carbon black does not contribute to the capacity. Therefore, the use of carbon black degrades the performance per unit weight. The superior conductivity and absence of conductive materials and binders allows LSG to exhibit higher energy and power densities^67^. It can be inferred that the LSG/H-HTO system is a powerful candidate for improving the electrochemical performances of hybrid supercapacitors.

## Conclusion

In summary, we designed novel compositions of LSG cathodes and H-HTO anodes for hybrid supercapacitors. The LSG/H-HTO combination clearly exhibits superior electrochemical activity with a cycling stability of 98% after 10000 cycles and a rate capability of 78%, even at a high current density of 3.0 A/g. In addition, the energy and power densities reach 17.7–70.8 Wh/kg and 195.1–5191.9 W/kg, respectively, which are higher than those of previously reported hybrid supercapacitors. As demonstrated, LSG possesses a higher effective surface area, ensuring greater energy storage as a high-energy cathode. Also, H-HTO provides rapid and stable pathways for fast lithium ion and electron transfer as a high-power anode. Considering the superior safety and excellent energy and power densities, we conclude that the electrode composition of an LSG cathode and an H-HTO anode holds great promise for future hybrid supercapacitors.

## Electronic supplementary material

Supplementary Information
