# Supplementary material for: Laser Scribed Graphene Cathode for Next Generation of High Performance Hybrid Supercapacitors
Source: Sci Rep. 2018 May 25;8:8179. doi: 10.1038/s41598-018-26503-4 (PMC5970221; doi:10.1038/s41598-018-26503-4)
Supplement: Supplementary file 1 — Supplementary Information [file 41598_2018_26503_MOESM1_ESM.doc]

Supporting Information

**Laser Scribed Graphene Cathode for Next Generation of**

**High Performance Hybrid Supercapacitors**

Seung-Hwan Lee,^a,1^,*, Jin Hyeon Kim^b,1^, Jung-Rag Yoon^c,*^

Author Affiliation and Department

^a^Institute for Research in Electronics and Applied Physics, University of Maryland, College Park, Maryland 20742, USA
 ^b^Dept. of Electronics Materials Engineering, Kwangwoon University, Seoul, Korea
^c^R&D center, Samwha Capacitor, Korea

Author(s) address
^a^ University of Maryland, Room 1103, Building 89, Colleage Park, MD 20742, USA
^b^ Kwangwoon Univ., Wolgye 1-dong, Nowon-gu, Seoul, Korea
^c^ Samwha Capacitor, 227 Gyeonggidong-ro, Namsa-myeon, Cheoin-gu, Yongin-si, Gyeonggi-do, Korea

Author(s) e-mail addresses

^1*^ [shlee83@umd.edu](mailto:shlee83@umd.edu)

^2*^ [yoonjungrag@samwha.com](mailto:yoonjungrag@samwha.com)

^1^These authors equally contributed to this work.


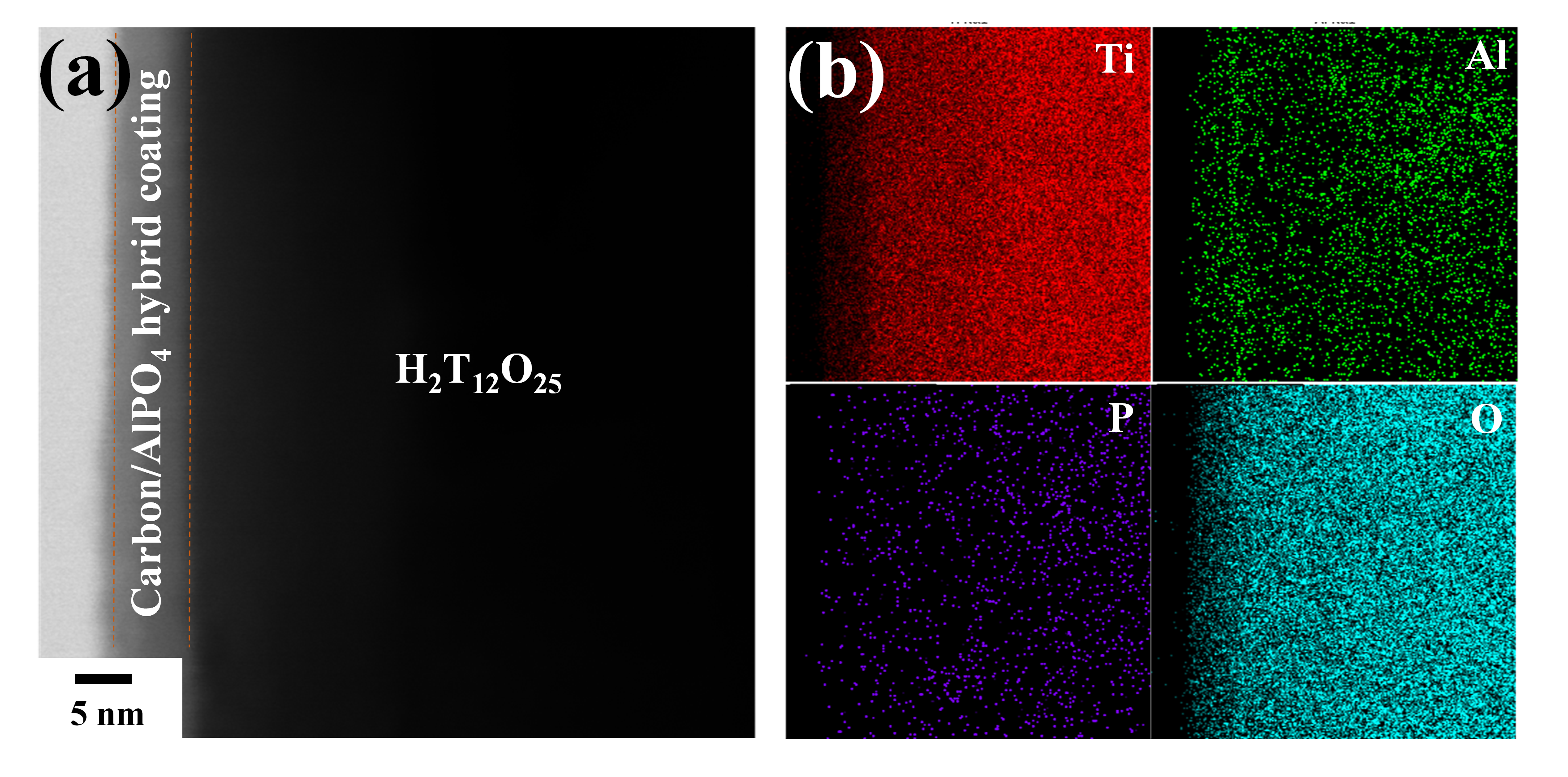


Figure S1†. (a) TEM image of H-HTO (b) EDS mapping of H-HTO


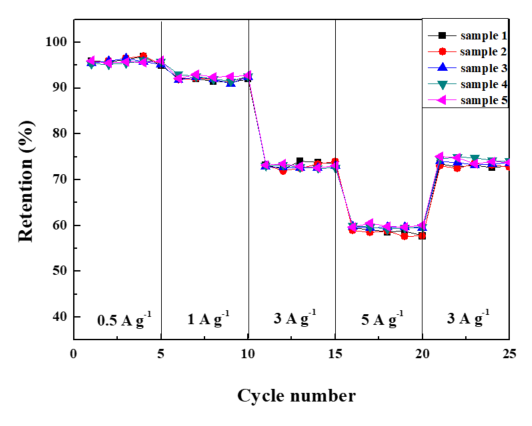


Figure S2†. Reproducibility of hybrid supercapacitor using LSG/H-HTO


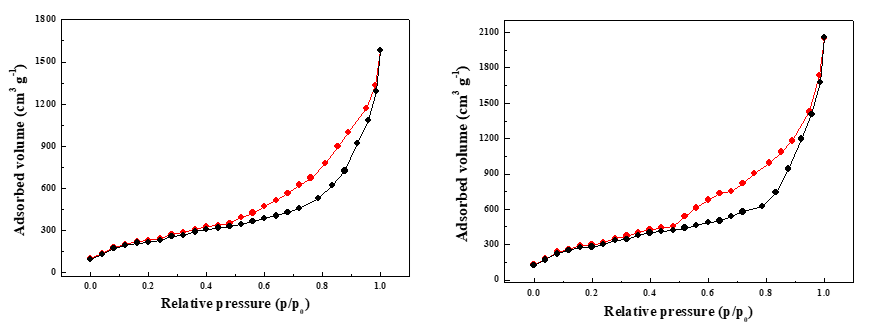


Figure S3†. Nitrogen adsorption/desorption isotherms of LSG
